# Effect of Black Tea and Black Tea Pomace Polyphenols on α-Glucosidase and α-Amylase Inhibition, Relevant to Type 2 Diabetes Prevention

**DOI:** 10.3389/fnut.2015.00003

**Published:** 2015-02-12

**Authors:** Lisa Striegel, Bouhee Kang, Sarah J. Pilkenton, Michael Rychlik, Emmanouil Apostolidis

**Affiliations:** ^1^Department of Chemistry and Food Science, Framingham State University, Framingham, MA, USA; ^2^Technische Universitaet Muenchen, Chair of Analytical Food Chemistry, Freising, Germany

**Keywords:** α-glucosidase inhibition, black tea, black tea pomace, α-amylase inhibition, phenolic compounds, type 2 diabetes, pre-diabetes

## Abstract

This study evaluates the potential mechanism of action and bioactivity of black tea and black tea pomace for type 2 diabetes prevention via inhibition of carbohydrate hydrolyzing enzymes. Black tea leaves were extracted in hot water and black tea pomace was extracted in 70% acetone. The phenolic content of the water extract (WBT) and pomace acetone extracts (AOBT) were 5.77 and 8.9 mg/mL, respectively, both based on the same concentration of solid tea in the extract. The water extract was subjected to C_18_ extraction and the resulting hydrophobic fraction (HBBT) was further subjected to LH-20 extraction to recover a low molecular weight phenolic enriched fraction (LMW) and a high molecular weight enriched fraction (HMW). The phenolic content of the LMW and HMW fraction were 1.42 and 2.66 mg/mL, respectively. Among water extracts the HMW fraction was most bioactive against α-glucosidase (IC_50_ = 8.97 μg/mL) followed by HBBT fraction (IC_50_ = 14.83 μg/mL). However, the HBBT fraction was the most bioactive fraction against α-amylase (IC_50_ = 0.049 mg/mL). The black tea pomace (AOBT) had significant α-glucosidase inhibitory activity (IC_50_ = 14.72 μg/mL) but lower α-amylase inhibitory activity (IC_50_ = 0.21 mg/mL). The phenolic profiles for LMW and HMW fractions were evaluated using HPLC and the differences between the two profiles were identified. Further research is underway to identify and evaluate the phenolic compounds that are present in the HMW fraction. Our findings suggest that black tea and black tea pomace has potential for carbohydrate hydrolyzing enzyme inhibition and this activity depends on high molecular weight phenolic compounds.

## Introduction

According to International Diabetes Federation (IDF) worldwide 382 million people in 2013 had diabetes and this number is projected to increase to 592 million by 2035 (1). This dramatic increase is mainly due to the increasing incidence of type 2 diabetes (1), also called non-insulin-dependent diabetes, which is caused by the body’s ineffective use of insulin (2). To prevent the increase of type 2 diabetes, Centers of Disease Control and Prevention (CDC) defined a state of borderline type 2 diabetic patients, as pre-diabetes. The American Diabetes Association defines pre-diabetic individual as an individual with blood glucose levels higher than normal (impaired fasting glucose between 100 and 125 mg/dL, impaired glucose tolerance between 140 and 199 mg/dL, and HbA1c between 5.7 and 6.4%) but not high enough to be considered diabetic (impaired fasting glucose between >126 mg/dL, impaired glucose tolerance between >200 mg/dL, and HbA1c between >6.5%) (3, 4).

Beside lifestyle change, which can prevent type 2 diabetes there are also food-based active ingredients, such as polyphenols, which have the ability to modulate blood glucose level (5). Recent research has shown that phenolic compounds have the potential to inhibit carbohydrate hydrolyzing enzymes in our digestive organs and thus have potential to play a role in management of type 2 diabetes (6).

The major source of glucose in our body comes from the hydrolysis of dietary carbohydrates, such as starch. The pancreatic alpha-amylase and intestinal alpha-glucosidases are the hydrolyzing enzymes responsible for glucose generation via diet (6). α-Amylase hydrolyzes alpha-1,4-glycocidic bonds and splits up starch components such as amylose and amylopectin into smaller oligosaccharides and disaccharides, like maltose. The α-glucosidases hydrolyze disaccharides to monosaccharides. Previous reports suggest that the inhibition of these enzymes can be an important concept for management of type 2 diabetes (7, 8). Additional studies have shown that α-glucosidase inhibitors, such as acarbose, are the only oral anti-diabetes agent approved for the treatment of pre-diabetes (9).

Tea is one of the most consumed beverages of the world. The tea plant (*Camellia sinensis*) from which the beverage tea is processed is a member of the genus *Camellia* and has over 200 species and is originated in the highlands of Tibet, north eastern India, and southern China. Today the major tea production countries are India, China, Sri Lanka, Kenya, Japan, Vietnam, Bangladesh, and Indonesia (10). Tea is generally consumed as green tea, oolong tea, or black tea (11). This classification is according to degree of fermentation: unfermented tea is green tea, semi-fermented tea is oolong tea, and fully fermented tea is black tea (10). The different process leads to unique characteristics, taste, and chemical profile. Of these three types, black tea is most widely produced and consumed, approximately 80%.

Polyphenols belong to phenolic phytochemicals and are secondary plant metabolites, widely distributed in plants. They are known to be strong antioxidants and hence they are able to prevent tissue damage caused by free radicals (10). The major phenolic compounds present in green tea are flavonols, and more specifically catechins (10). Compared to green tea, the catechin content in black tea is reduced by approximately 85% and transformed into teaflavin-3-3′-digallate and thearubigin, which are catechin polymerization products (10). Previous research suggests that these compounds can retard the absorption of glucose by inhibition of carbohydrate hydrolyzing enzymes, such as α-glucosidase and α-amylase in the digestive organs. Hence, α-glucosidase inhibitors can retard the liberation of glucose from dietary complex carbohydrates and delay glucose absorption, resulting in reduced postprandial plasma glucose levels and suppression of postprandial hyperglycemia. This could be a good treatment for type 2 diabetes prevention (12). Specifically for tea, Kwon et al. (6) reported that green tea had the lowest α-glucosidase inhibitory activity, followed by Oolong tea and black tea had the highest inhibitory activity. These findings suggest that the observed α-glucosidase inhibitory activity possibly depends on the catechin polymerization products that are produced during fermentation.

In this study, we evaluate the potential mechanism and bioactives of black tea and black tea pomace for type 2 diabetes prevention via inhibition of carbohydrate hydrolyzing enzymes. More specifically, we focus on the evaluation of black tea water extract polyphenols and investigate if the observed bioactivity is low molecular weight phenolic dependent (i.e., catechins) or high molecular weight phenolic dependent (i.e., catechin polymerization products). Additionally, we extract the resulting pomace using acetone and briefly evaluate its potential for carbohydrate hydrolyzing enzyme inhibition. Completion of this study gives a better understanding of the possible effect and mechanism of black tea and black tea pomace for possible prevention of type 2 diabetes.

## Materials and Methods

### Chemical/materials used

The black tea used in our experiment (Lipton – Earl Grey bergamot flavor) was purchased from a local supermarket (Stop and Shop, Framingham, MA, USA). α-Glucosidase from *Saccharomyces cerevisiae* (EC 3.2.1.20) as well as porcine pancreatic α-amylase (EC 3.2.1.1) were purchased from Sigma-Aldrich Co. (St. Louis, MO, USA). Unless noted, all chemicals were also purchased from Sigma-Aldrich Co. (St. Louis, MO, USA). All solvents used were HPLC grade.

### Sample preparation

Initially, 7.5 g tea leaves were extracted in 75 ml of distilled water for 30 min at 90°C. After extraction the resulting sample was filtered through a Whatman #54 to give the initial working sample (WBT). WBT was stored at 4°C until further testing. The resulting pomace was collected from the filter and was extracted in 70% acetone. Briefly, leaves were dried in a drying oven at 100°C (AOBT). After drying, 6 g of the dried leaves were extracted in 60 mL of a 70% acetone solution for 2 h at room temperature and under constant stirring. The resulting extract was filtered using a Whatman #54 filter and stored at 4°C until further testing.

### C_18_ cartridge extraction

The water extract (20 mL of WBT) was subjected to solid phase extraction using C_18_ cartridge (DSC-18, 3 mL Tube, 500 mg; Supelco, Bellefonte, PA, USA). A subsample (500 μL) of the extract was loaded onto each pre-conditioned C_18_ (acetone followed by distilled water) followed by rinsing with deionized water to remove the hydrophilic compounds (HPBT). After washing, the hydrophobic compounds (including phenolic compounds) were eluted with 5 mL of 99:1 (v/v) methanol:acetic acid solution (HBBT). The solvent and water were evaporated from HBBT and HPBT, respectively, using a rotary evaporator (Fisher Scientific; Hanover Park, IL, USA) at 60°C to yield a final volume of 20 mL. The evaporated extract was stored at −4°C until further testing.

### LH-20 column extraction

The hydrophobic fraction (HBBT) was subjected to gel filtration chromatography using Sephadex LH-20 column extraction to recover a high molecular weight fraction (HMW) and a low molecular weight fraction (LMW) of phenolic compounds. Three milliliter of hydrophobic fraction was loaded onto 40 mL LH-20 column, which has been conditioned by washing with 200 mL of 30% methanol. The column was washed with 600 mL 30% methanol to recover low molecular weight phenolic compounds (LMW) and with 100 mL of acetone: deionized water: acetic acid (70:29.9:0.01) (v/v/v) to collect the high molecular weight phenolic compounds (HMW). A total of 20 mL of HBBT was subjected to LH-20 extraction in portions of 2 and 3 mL and finally both fractions were roto-evaporated by vacuum rotor evaporator (Fisher Scientific; Hanover Park, IL, USA) to 20 mL to remove solvents and stored at 4°C until further testing.

### Total phenolic content determination

The phenolic content of all extracts was determined by an assay modified from Shetty et al. (13). Briefly, 0.5 mL of sample or standard was mixed with 0.5 mL distilled water, 1 mL 95% ethanol, 5 mL distilled water, and 0.5 mL 50% (v/v) Folin–Ciocalteu reagent in test tubes. Then the test tubes were incubated at room temperature for 5 min. After adding of 1 mL of 5% Na_2_CO_3_, the mixtures were kept in the dark at room temperature for 60 min. The absorbance was measured at 725 nm (U 2001 Spectrophotometer; Hitachi, Pleasanton, CA, USA) after vortexing. Gallic acid standards were prepared (15.625, 31.25, 62.5, 125, 250, and 500 μg/mL in ethanol) and used to establish the standard curve. Results were expressed as mg of gallic acid equivalents per gram of sample (DW).

### Antioxidant activity by 1,1-diphenyl-2-picrylhydrazyl radical inhibition assay

The antioxidant activity by 1,1-Diphenyl-2-picrylhydrazyl (DPPH) free-radical scavenging inhibition was determined following the procedure modified from Shetty et al. (13). The percentage antioxidant activity (AA%) was determined in a dose-dependent manner. Stock DPPH solution was prepared by adding 39 mg DPPH in 100 mL 95% ethanol. To 1 mL of DPPH solution 200 μL of each extract was added and the decrease in absorbance was monitored at 517 nm, using a spectrophotometer (U 2001 Spectrophotometer; Hitachi, Pleasanton, CA, USA) until a constant reading was obtained. The readings were compared with the controls, which contained 200 μL of water instead of the extract. The % inhibition was calculated by:

$$\%\;\mathrm{inhibition =}\left(\frac{{\mathrm{Abs}}_{\mathrm{control}}-{\mathrm{Abs}}_{\mathrm{sample}}}{{\mathrm{Abs}}_{\mathrm{control}}}\right)\times 100$$

### Yeast-α-glucosidase inhibition assay

The dose-dependent yeast α-glucosidase inhibitory activity was determined in all whole extracts and recovered hydrophilic and hydrophobic fractions after C_18_ extraction. A mixture of 50 μL extract and 100 μL of 0.1 M phosphate buffer (pH 6.9) containing α-glucosidase solution (1.0 U/mL) were incubated in 96 well plates at 25°C for 10 min. After pre-incubation, 50 μL of 5 mM p-nitrophenyl-α-d-glucopyranoside solution in 0.1 M phosphate buffer (pH 6.9) was added to each well at timed intervals. The reaction mixtures were incubated at 25°C for 5 min. Before and after incubation, absorbance was recorded at 405 nm by micro-plate reader (VMax, Molecular Device Co., Sunnyvale, CA, USA) and compared to that of the control, which had 50 μL buffer solution in place of the extract. The α-glucosidase inhibitory activity was expressed as inhibition % and was calculated as followed:

$$\%\;\mathrm{inhibition =}\left(\frac{\Delta {\mathrm{Abs}}_{\mathrm{control}}-\Delta {\mathrm{Abs}}_{\mathrm{sample}}}{\Delta {\mathrm{Abs}}_{\mathrm{control}}}\right)\times 100$$

### Porcine α-amylase inhibition assay

The dose-dependent α-amylase inhibitory activity of water extracts was evaluated. A mixture of 500 μL extract and 500 μL 0.02 M sodium phosphate buffer (pH 6.9 with 0.006 M sodium chloride) containing α-amylase solution (13 U/mL) was incubated at 25°C for 10 min. After pre-incubation, 500 μL of 1% soluble starch solution in 0.02 M sodium phosphate buffer (pH 6.9 with 0.006 M NaCl) was added to each tube at timed intervals. The reaction mixtures were then incubated at 25°C for 10 min followed by addition of 1 mL dinitrosalicylic acid color reagent (14). The test tubes were then placed in a boiling water bath for 5 min to stop the reaction and cooled to room temperature. The reaction mixture was then diluted with 10 mL of distilled water and the absorbance was read at 540 nm. The % inhibitory activity was determined using the following equation:

$$\%\;\mathrm{inhibition =}\left(\frac{\Delta {\mathrm{Abs}}_{\mathrm{control}}-\Delta {\mathrm{Abs}}_{\mathrm{sample}}}{\Delta {\mathrm{Abs}}_{\mathrm{control}}}\right)\times 100$$

### Phenolic profile determination with HPLC

The obtained HBBT, HMW, and LMW fractions were subjected to HPLC to identify the differences in their phenolic profile. All tested samples were concentrated by rotary evaporation (Fisher Scientific; Hanover Park, IL, USA) to complete dryness and were re-dissolved in 1 mL of acetone. To 800 μL of each sample we added 400 μL of distilled water and the resulting solutions were filtered using a 0.45 μm Fisherbrand^®^ syringe filter (Thermo Fisher Sci, Pittsburg, PA, USA). An injection volume of 20 μL of each sample was injected and analyzed using a reverse phase C-18 column (Agilent ZORBAX Extend C-18 column, 250 × 4.6 mm id.d, 5 μm particle size). The mobile phase consisted of solvent A (4% phosphoric acid) and solvent B (acetonitrile). Gradient elution was used under linear gradient conditions starting with 95% A and decreasing linearly to 65% A over a 70 minute time period at a flow rate of 0.75 mL/min. Phenolic profiles were observed at 254 nm and 280 nm using a UV-VIS detector.

### Statistical analysis

All experiments were performed at least three times in triplicates. Analysis at every time point from each experiment was carried out in triplicates. Means, standard errors, standard deviations, and IC_50_ values were calculated using Microsoft Excel XP.

## Results

### Total phenolic content

**Figure 1:**
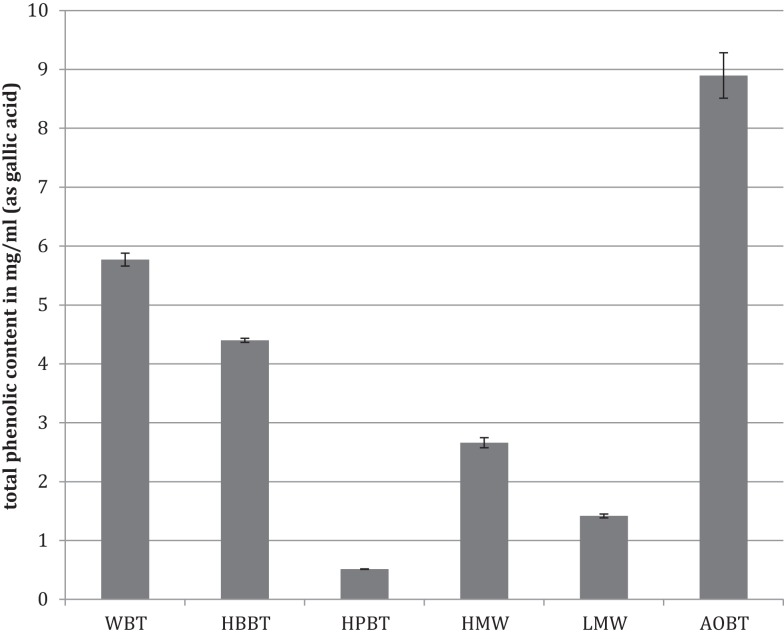
**Total phenolic content of black tea water extract fractions and black tea pomace**.

The total phenolic content analyzed by Folin–Ciocalteau’s method indicated that WBT had a phenolic content of 5.77 mg/mL (Figure 1). As expected the total phenolic content of HBBT was higher than the total phenolic content of HPBT (4.4 and 0.52 mg/mL, respectively) (Figure 1). After LH-20 extraction of HBBT fraction the total phenolic content of HMW and LMW were determined to be 2.66 and 1.43 mg/mL, respectively (Figure 1).

The WBT fraction had the highest phenolic content followed by the HBBT fraction, which resulted after the C_18_ extraction. The HBBT fraction was further subjected to LH-20 column separation resulting in HMW and LMW fraction. HMW fraction had higher phenolic content and the sum of both HMW and LMW phenolic contents was similar to the HBBT phenolic content, suggesting a successful recovery of phenolic phytochemicals following LH-20 extraction. This shows that all phenolic compounds initially present in the HBBT fraction were eluted either in methanol or in acetone.

The phenolic content of pomace acetone extract (AOBT) was 8.63 mg/mL (Figure 1), suggesting that black tea pomace resulting from hot water black tea extraction contains significant amount of phenolic compounds that cannot be extracted using water as an extraction medium.

### Antioxidant activity by DPPH radical inhibition assay

**Figure 2:**
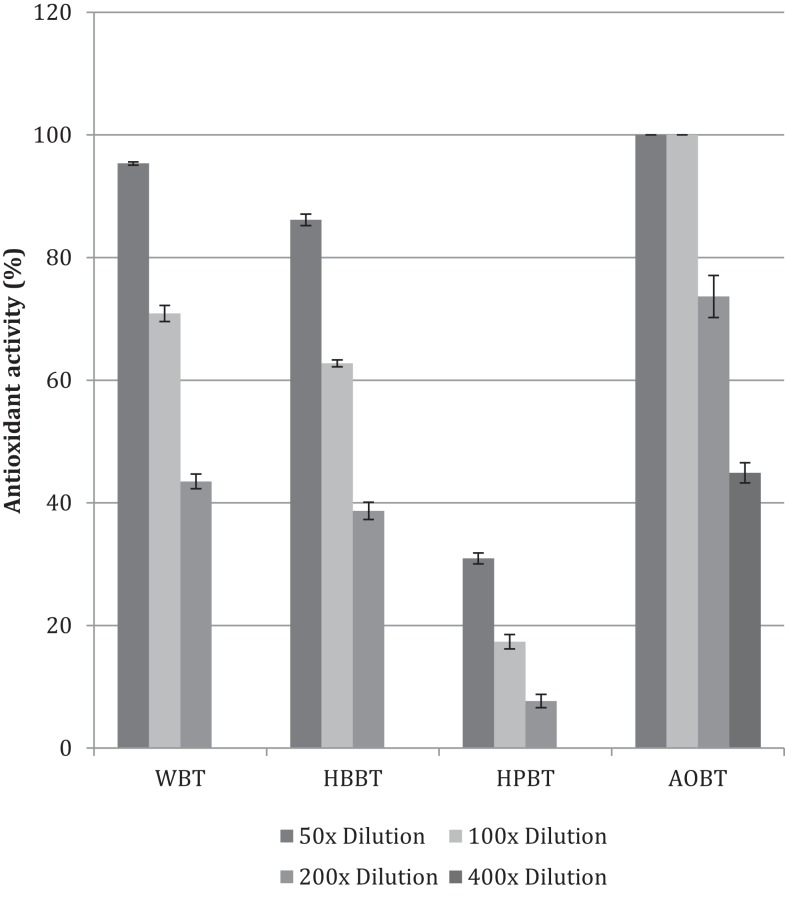
**Antioxidant activity of black tea water extract fractions and black tea pomace**.

The antioxidant activity in terms of DPPH free-radical scavenging potential was evaluated for WBT, HBBT, and HPBT (Figure 2). All tested samples had antioxidant activity in a dose-dependent manner in the tested doses. More specifically, WBT fraction had the highest antioxidant activity with 95.4% radical inhibition (50× dilution) followed by HBBT (86.2% at the same dilution) (Figure 2). HPBT fractions had the lowest antioxidant activity (30.9% at the same dilution) (Figure 2). When the black tea pomace acetone extract (AOBT) was evaluated we determined that it had the highest antioxidant activity (100% at 50× dilution) (Figure 2).

It is well documented that phenolic phytochemicals are effective antioxidants (6, 15–18) and our findings indicate that the antioxidant activities are in agreement with the phenolic contents (Figures 1 and 2).

### α-Glucosidase inhibition assay

The dose-dependent α-glucosidase inhibitory activities of WBT, HBBT, HPBT, LMW, HMW, and AOBT were investigated. For comparison, the results were expressed in IC_50_ values based on the phenolic content (the phenolic concentration of extracts resulting in 50% inhibition of enzyme activity). We observed that the HMW fraction appeared to be the most bioactive fraction (IC_50_ 8.97 μg phenolics/mL), followed by the HBBT fraction, with an IC_50_ of 14.8 μg phenolics/mL (Table 1). The IC_50_ of the WBT and LMW fractions were calculated to be 22.4 and 34.2 μg phenolics/mL, respectively, followed by HPBT (IC_50_: 503 μg/mL), which had the lowest inhibitory activity (Table 1).

**Table 1:** **Yeast α-glucosidase inhibition IC_50_ values based on total phenolic content**.

	WBT	HBBT	HPBT	LMW	HMW	AOBT
IC_50_ (μg/mL)	22.4	14.8	502	34.2	8.97	14.7

Our observations clearly suggest that the most bioactive components in black tea for α-glucosidase inhibition are high molecular weight phenolic compounds. Our observations are in agreement with our preliminary observations that the α-glucosidase inhibitory activity increases as tea is fermented (6). During fermentation low molecular weight catechins are polymerized to form higher molecular weight teaflavin-3-3′-digallate and thearubigin, which are catechin polymerization products (10).

When the α-glucosidase inhibitory activity of AOBT was evaluated we observed an IC_50_ 14.7 μg phenolics/mL (Table 1). This inhibitory activity is lower than HBBT and HMW than the whole black tea water extract (WBT). Our findings suggest that the black tea pomace contains hydrophobic compounds that are not extracted during hot water extraction, which have significant potential for type 2 diabetes prevention via inhibition of carbohydrate hydrolyzing enzymes. We suspect that these compounds are hydrophobic in nature and most probably higher molecular weight catechin polymerization products.

### α-Amylase inhibition assay

Similarly to the α-glucosidase inhibitory activities, the IC_50_ values of the α-amylase inhibitory activities were estimated for all samples (Table 2). The IC_50_ of the WBT fraction was 1.74 mg phenolics/mL, while the inhibitory activity of the HBBT fraction was the most bioactive of all the tested fractions with an IC_50_ of 0.049 mg phenolics/mL. The HPBT fraction had a negligible IC_50_ (131 mg phenolics/mL), while the IC_50_ of the HMW fraction was calculated to be 0.42 mg phenolics/mL. Finally, AOBT extract resulted to an IC_50_ value of 0.21 mg phenolics/mL.

**Table 2:** **α-Amylase inhibition IC_50_ values based on total phenolic content**.

	WBT	HBBT	HPBT	LMW	HMW	AOBT
IC_50_ (mg/mL)	1.74	0.049	131	–	0.42	0.21

After comparing the resulting α-glucosidase and α-amylase IC_50_ values the α-amylase inhibitory activity of the testes samples was lower than the α-glucosidase inhibitory activity (Tables 1 and 2). The main side effects of type 2 diabetes control drugs, such as acarbose, are abdominal distention, flatulence, meteorism, and possibly diarrhea (19). It has been suggested that such adverse effects might be caused by the excessive inhibition of pancreatic α-amylase resulting in the abnormal bacterial fermentation of undigested carbohydrates in the colon (19). In our study, although we observed a low α-amylase inhibitory activity, the possibility of reduced side effects linked to excessive inhibition of starch digestion is suggested.

### Phenolic profile determination with HPLC

**Figure 3:**
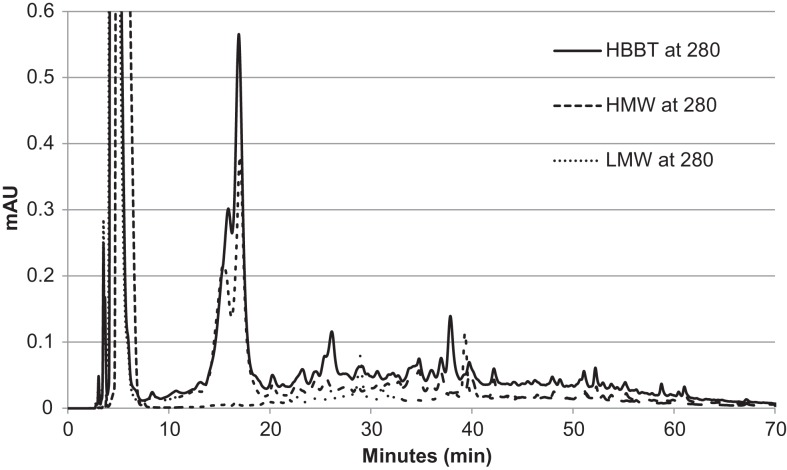
**HPLC chromatogram of HBBT, HMW, and LMW at 280 nm**.

**Figure 4:**
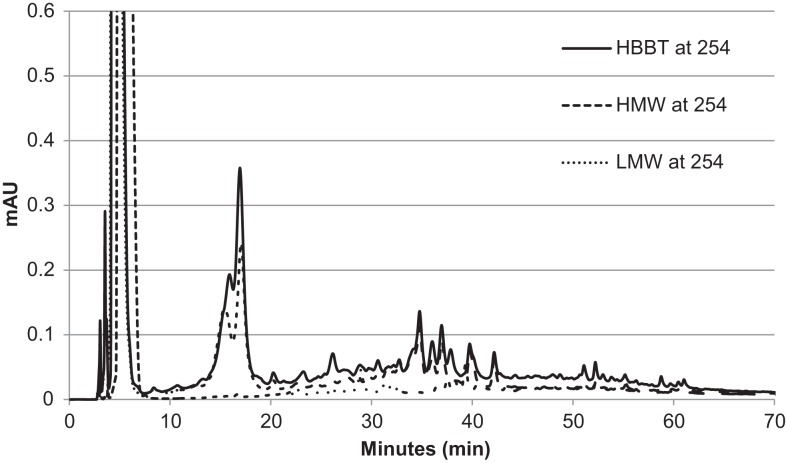
**HPLC chromatogram of HBBT, HMW, and LMW at 254 nm**.

To confirm the successful fractionation using LH-20 extraction, the phenolic profiles of HBBT, LMW, and HMW were determined at 254 and 280 nm as described in the Section “Materials and Methods” (Figures 3 and 4). Our observations suggest a different phenolic profile in the three tested samples. The HBBT and LMW fraction showed two peaks after 15.6 and 16.7 min with λ_max_ at 280 nm (Figure 3). These peaks were absent in the HMW fraction. Additionally, at 34.7, 35.8, and 36.8 min the HBBT and LMW fractions showed peaks with λ_max_ at 254 nm (Figure 4). There were also peaks at 39.7 and 42.1 min with a λ_max_ at 254 nm found in the HBBT and LMW fractions (Figure 4). These findings suggest that these peaks are possibly low molecular weight phenolic compounds. We were also able to identify unique peaks found only in the HBBT and HMW fraction. We noticed that the HMW chromatogram was slightly shifted, when compared to the HBBT. In the HBBT fraction, two peaks were identified at 25.0 and 25.8 min. The same peaks could be identified in the HMW fraction after 28.6 and 28.9 min. Additionally, in HBBT a peak was identified at 37.7 min and this peak was also detected in the HMW fraction at 39.2 min. Both peaks had a λ_max_ at 280 nm (Figure 3). Furthermore, three more peaks were identified to be unique for the HBBT and HMW fraction. These compounds eluted at 47.4, 58.7, and 70.0 min and had a λ_max_ at 280 nm (Figure 3) and could not be identified in the LMW fraction.

Our results suggest that LH-20 extraction of HBBT using two different solvents yielded two different fractions (LMW and HMW) that have different phenolic profile. Although no compounds were characterized in this research, we suspect that the peaks appearing in both HBBT and HMW are high molecular weight catechin polymerization products. More research is underway to effectively characterize the resulting HMW fraction and link the identified compounds with specific carbohydrate hydrolyzing enzyme inhibitory activity.

## Conclusion

Type 2 diabetes is an emerging health concern in many parts of the world. It is projected that diabetes will increase dramatically during the next years and will be the 7th leading cause of death in 2030, mainly due to the increase in the incidence of type 2 diabetes (1, 2).

It is well documented that phenolic phytochemicals have α-glucosidase inhibitory activity that depends on the phenolic profile (6, 17, 20). A recent study reported that cinnamon extract has α-glucosidase inhibitory activity, which is low molecular weight phenolic dependent (20). Another study in our lab showed that the α-glucosidase inhibitory activity of blueberries is low molecular weight phenolic dependent (21). In the case of blueberries and cinnamon, the predominant high molecular weight phenolic compounds are proanthocyanidins.

In this preliminary work, we identified that both black tea water extract and black tea pomace have potential for type 2 diabetes prevention, via inhibition of carbohydrate hydrolyzing enzymes, namely α-glucosidase. The same extracts have a low α-amylase inhibitory activity. Strong inhibition of α-glucosidase and low inhibition of α-amylase could be potentially used as an effective complementary therapy for postprandial hyperglycemia linked to type 2 diabetes with reduced side effects. Our fractionation results, coupled with the high bioactivity of the black tea pomace, suggest that the observed effects are probably due to high molecular weight phenolics present in black tea, namely catechin polymerization products. This conclusion is further supported from our previous findings (6) that suggest that α-glucosidase inhibition in tea increases during fermentation. Further investigation is underway to identify the specific high molecular weight phenolic compounds in black tea and pomace of water extraction that are relevant to the inhibition of carbohydrate hydrolysis enzymes.

## Author Contributions

LS, BK, and SP carried out tea extraction, fractionization, and the inhibition assays. The study was designed by EA. The manuscript was written by LS, MR, and EA.

## Conflict of Interest Statement

The authors declare that the research was conducted in the absence of any commercial or financial relationships that could be construed as a potential conflict of interest.

## Abbreviations

AOBT, acetone extract black tea; HBBT, hydrophobic fraction from black tea water extract; HMW, high molecular phenolic-containing fraction (resulting from HBBT); HPBT, hydrophilic fraction from black tea water extract; LMW, low molecular weight phenolic-containing fraction (resulting from HBBT); WBT, water extract black tea.
